# Up and About: Older Adults’ Well-being During the COVID-19 Pandemic in a Swedish Longitudinal Study

**DOI:** 10.1093/geronb/gbaa084

**Published:** 2020-06-30

**Authors:** Marie Kivi, Isabelle Hansson, Pär Bjälkebring

**Affiliations:** 1 Department of Psychology, University of Gothenburg, Sweden; 2 Centre for Ageing and Health, University of Gothenburg, Sweden

**Keywords:** Longitudinal change, Mental health, Risk perception

## Abstract

**Objectives:**

To investigate early effects of the COVID-19 pandemic related to (a) levels of worry, risk perception, and social distancing; (b) longitudinal effects on well-being; and (c) effects of worry, risk perception, and social distancing on well-being.

**Methods:**

We analyzed annual changes in four aspects of well-being over 5 years (2015–2020): life satisfaction, financial satisfaction, self-rated health, and loneliness in a subsample (*n* = 1,071, aged 65–71) from a larger survey of Swedish older adults. The 2020 wave, collected March 26–April 2, included measures of worry, risk perception, and social distancing in response to COVID-19.

**Results:**

(a) In relation to COVID-19: 44.9% worried about health, 69.5% about societal consequences, 25.1% about financial consequences; 86.4% perceived a high societal risk, 42.3% a high risk of infection, and 71.2% reported high levels of social distancing. (b) Well-being remained stable (life satisfaction and loneliness) or even increased (self-rated health and financial satisfaction) in 2020 compared to previous years. (c) More worry about health and financial consequences was related to lower scores in all four well-being measures. Higher societal worry and more social distancing were related to higher well-being.

**Discussion:**

In the early stage of the pandemic, Swedish older adults on average rated their well-being as high as, or even higher than, previous years. However, those who worried more reported lower well-being. Our findings speak to the resilience, but also heterogeneity, among older adults during the pandemic. Further research, on a broad range of health factors and long-term psychological consequences, is needed.

The COVID-19 pandemic is a major threat to public health, and older adults are particularly vulnerable for severe health consequences (Le Couteur et al., 2020; Verity et al., 2020; Zhou et al., 2020). As illustrated by the COVID-19 Government Response Stringency Index (Hale et al., 2020), responses to COVID-19 have varied along a continuum, with some imposing lockdowns, while others rely on guidelines and recommendations to slow the spread. Close to one end of this continuum, Sweden has largely remained open, relying on voluntary measures and imposing comparably few mandatory restrictions. The impact on daily life has nevertheless been huge, especially for those older than 70 years. The stated aim of many government responses to COVID-19 has been to protect older adults and other risk groups. However, lockdowns and arbitrary age restrictions may also put additional strain on older adults (Ayalon et al., 2020). The psychological consequences related to the pandemic itself, but also to the varying governmental responses to the pandemic, are still relatively unknown. Due to the heterogeneity of responses, a broad range of studies, from a variety of places, will be needed to fully understand the consequences for older adults.

The present study investigates well-being in a Swedish sample during the early days of the COVID-19 pandemic. The first case of COVID-19 in Sweden was reported February 1 (World Health Organization [WHO], 2020a). On March 25, Swedish authorities issued statements recommending social distancing, but did not enact a general lockdown. An additional, yet voluntary, recommendation to “shelter in place” was also directed to adults aged 70 and older (Public Health Agency of Sweden, 2020a, 2020b, 2020c). At the time of data collection, Sweden had 28 confirmed deaths per million (12th highest death rate) and the number of infected doubled every 3 days (WHO, 2020b).

In times of crisis, protecting lives and physical health is paramount; nevertheless, accurate assessment and understanding of psychological health should guide governmental response (Drury et al., 2013). WHO has warned of lower well-being during the pandemic, especially among older adults (WHO, 2020c). In the United States, worry has risen and well-being is at a 12-year low (Witters & Harter, 2020). At the same time, one recent study suggests that older adults are less vulnerable to psychological distress and loneliness during the pandemic (Losada-Baltar et al., 2020). In order to assess the true impact of COVID-19, longitudinal studies are needed to compare well-being before, throughout, and after the pandemic has ended.

Our study uses data from the longitudinal HEalth, Aging and Retirement Transitions in Sweden (HEARTS) project to investigate the early psychological effects of COVID-19 in a sample of older adults. The HEARTS data provide a unique opportunity to investigate longitudinal effects on well-being over a period of 5 years (2015–2020). We specifically aim to: (a) determine levels of worry, risk perception, and social distancing in relation to COVID-19; (b) investigate longitudinal effects on life satisfaction, financial satisfaction, self-rated health, and loneliness; and (c) quantify the effects of worry, risk perception, and social distancing on well-being.

## Method

Since 2015 (from March to June each year), the HEARTS study has conducted an annual survey in a population-based sample (*N* = 5,913) of older adults born 1949–1955 (age 60–66 at baseline; for more info on HEARTS, please see Lindwall et al., 2017). Ethical approval for the HEARTS study was granted from the regional ethical approval board at the University of Gothenburg (Dnr: 970-14).

In this study we used a subsample of HEARTS participants who responded during the first 7 days of data collection in 2020 (March 26, 10 a.m., until April 2, 10 a.m.; *n* = 1,071). The 2020 questionnaire included an additional set of questions related to the COVID-19 pandemic. These questions assessed levels of worry in relation to health, financial, and societal consequences, perceived risk of societal consequences, likelihood of being infected, and social distancing. Further, participants indicated if they or anyone in their immediate surroundings had been diagnosed with COVID-19. Longitudinal data on life satisfaction (Diener et al., 1985), financial satisfaction, self-rated health, and loneliness (Russell et al., 1980) were included to account for changes in relation to previous measurements. Age, gender, education, and retirement status were included to control for sociodemographic differences. For measurement details on study items, see Supplementary Table S1.

Data were analyzed with linear mixed-effects models using the lme4 (Bates et al., 2015) package in R (R Core Team, 2019). Deviations in life satisfaction, financial satisfaction, self-rated health, and loneliness were analyzed by comparing the scores of the 2020 assessment to the scores across previous waves (2015–2019). The model included a linear slope (measurement year) centered on the 2020 wave, as well as a dummy variable separating the 2020 measurement (1) from previous years (0). Effects of worry, risk perception, and social distancing were evaluated in a second model by regressing the four well-being measures on each of the five predictor variables. The four well-being measures were analyzed separately; age, gender, education, and retirement status were included as control variables in all models. The alpha level was restricted to 0.0125 using the Bonferroni correction to account for multiple testing.

## Results

Respondents to the 2020 survey had a mean age of 68.1 years (*SD* = 2.0; range 65–71), 47.3% were women, 52.4% had tertiary education. The sample comprises more men and a larger proportion of individuals with tertiary education compared to the baseline sample of HEARTS participants (45.4% and 40.2%, respectively). About 2% (*n* = 22) of participants reported that they or someone in their immediate surrounding had (or previously had) a confirmed COVID-19 infection. An additional 6.6% (*n* = 71) said they had reason to believe that they or someone in their immediate surroundings had been infected. Descriptive statistics on the study variables can be found in Table 1; bivariate correlations among the four well-being measures (*r* = 0.24–0.51) are presented in Supplementary Table S2.

**Table 1.** Descriptive Statistics of the Study Variables by Year for the HEARTS Subsample of 1,071 Persons Surveyed in 2020

	Measurement year					
	2015	2016	2017	2018	2019	2020
*n* ^a^	1,071	1,036	1,028	1,025	1,034	1,071
Age, *M* (*SD*)	63.12 (1.99)	64.14 (1.99)	65.13 (1.99)	66.15 (1.99)	67.13 (2.00)	68.12 (1.99)
Gender, % women	47.34	48.17	48.15	47.42	47.49	47.34
Education, % tertiary	52.38	52.61	52.33	52.49	52.42	52.38
Retirement status, % working	78.15	69.21	58.56	48.59	41.78	34.45
Life satisfaction (range 1–7), *M* (*SD*)	4.97 (1.27)	4.98 (1.27)	5.08 (1.27)	5.05 (1.35)	5.12 (1.30)	5.16 (1.26)
Financial satisfaction (range 1–5), *M* (*SD*)	—	3.92 (0.92)	3.92 (0.92)	3.89 (0.91)	3.90 (0.94)	3.97 (0.90)
Self-rated health (range 1–6), *M* (*SD*)	4.83 (0.93)	4.81 (0.93)	4.79 (0.92)	4.75 (0.94)	4.70 (0.94)	4.83 (0.88)
Loneliness (range 1–4), *M* (*SD*)	1.46 (0.59)	1.46 (0.61)	1.45 (0.59)	1.46 (0.59)	1.43 (0.60)	1.44 (0.59)
Health worry (range 1–5), *M* (*SD*)	—	—	—	—	—	3.51 (1.10)
Societal worry (range 1–5), *M* (*SD*)	—	—	—	—	—	4.06 (0.95)
Financial worry (range 1–5), *M* (*SD*)	—	—	—	—	—	2.77 (1.25)
Societal risk (range 1–5), *M* (*SD*)	—	—	—	—	—	4.50 (0.79)
Risk of being infected (range 1–4), *M* (*SD*)	—	—	—	—	—	2.38 (0.71)
Social distancing (range 1–5), *M* (*SD*)	—	—	—	—	—	4.10 (0.97)

*Notes*: HEARTS = HEalth, Aging and Retirement Transitions in Sweden; *SD* = standard deviation.

^a^Not all respondents have responded to all surveys; thus, *n* differs slightly across measurement waves.

### Aim 1: To Determine Levels of Worry, Risk Perception, and Social Distancing in Relation to COVID-19

The results showed that 44.9% (i.e., proportion of individuals with a score ≥4) worried about their own or others’ health, 69.5% worried about societal consequences, and 25.1% worried about financial consequences related to COVID-19. The majority (86.4%) reported high societal risks, 42.3% perceived the risk of being infected as high (≥3), and 71.2% reported engaging in social distancing.

**Figure 1.**
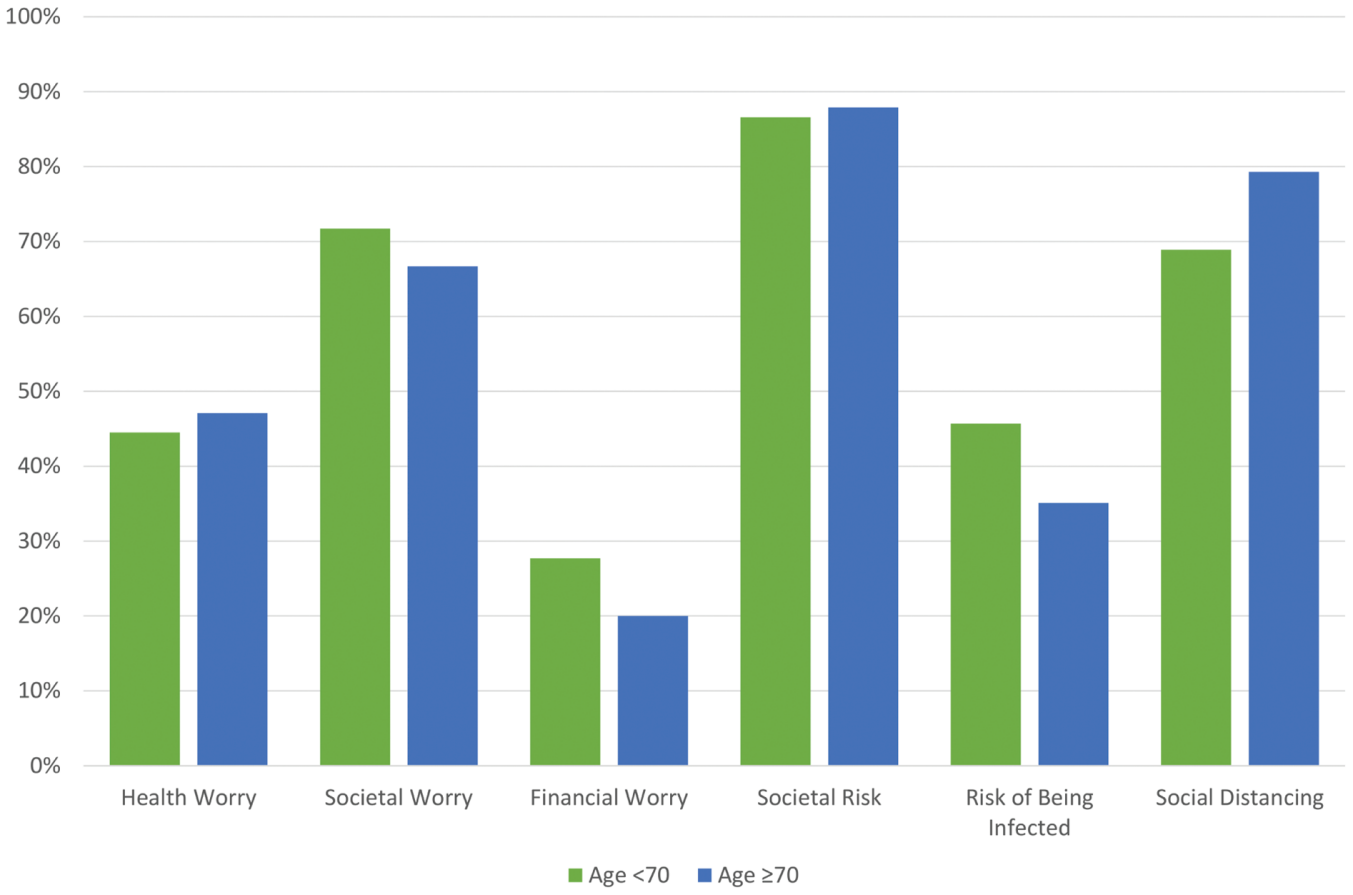
Proportion of individuals with high scores on level of (health, societal, and financial) worry (≥4), societal risk (≥4), risk of being infected (≥3), and social distancing (≥4), stratified on age groups (<70 and ≥70).

Figure 1 shows the proportion of individuals with high scores on level of worry, risk perception, and social distancing for individuals below and above age 70 (i.e., defined as a risk group by Swedish authorities; Public Health Agency of Sweden, 2020c). Participants aged 70 and older (*n* = 333) reported less financial worry (<70 = 27.7%, ≥70 = 20.0%; χ ^2^ = 6.73, *p* = .010), more social distancing (<70 = 68.9%, ≥70 = 79.3%; χ ^2^ = 11.81, *p* = .001), and a lower risk of being infected (<70 = 45.7%, ≥70 = 35.1%; χ ^2^ = 10.01, *p* = .002) compared to those aged 65–69. No significant differences were found with respect to health (<70 = 44.5%, ≥70 = 47.1%; χ ^2^ = 0.55, *p* = .46), societal worry (<70 = 71.7%, ≥70 = 66.7%; χ ^2^ = 2.51, *p* = .11), or societal risks (<70 = 86.6%, ≥70 = 87.9%; χ ^2^ = 0.24, *p* = .63).

### Aim 2: To Investigate Longitudinal Effects on Life Satisfaction, Financial Satisfaction, Self-Rated Health, and Loneliness

**Figure 2.**
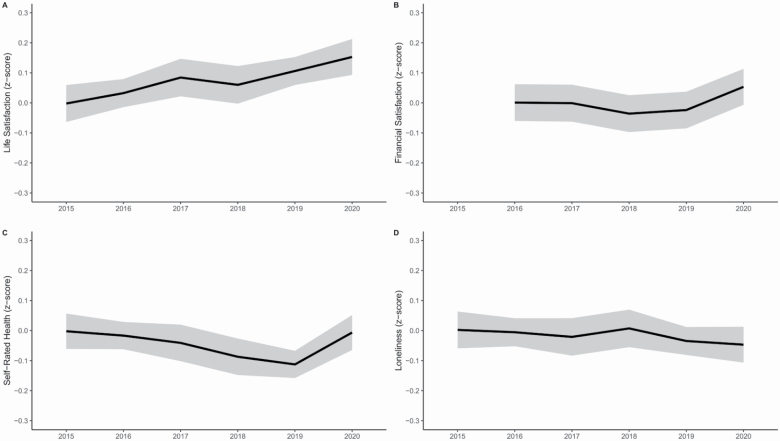
Yearly changes (2015–2020) in the four well-being domains: life satisfaction (A) showed a marginal but nonsignificant increase over time, financial satisfaction (B) was stable over time but increased in 2020, self-rated health (C) declined between 2015 and 2019 but increased in 2020, and loneliness (D) showed no change across years.

While self-rated health declined over time (β = −0.05, *p* < .001), it was higher in the 2020 wave compared to previous assessments (β = 0.18, *p* < .001), indicating that perceived health in 2020 is comparable to 5 years earlier. Financial satisfaction was stable across time (β = 0.002, *p* = .79), but was significantly higher in 2020 compared to all previous years (β = 0.09, *p* < .001). Life satisfaction (β = 0.01, *p* = .015) and loneliness (β = −0.003, *p* = .69) were stable over time with no significant deviation in the 2020 wave (life satisfaction: β = 0.02, *p* = .32; loneliness: β = −0.01, *p* = .84). Figure 2 illustrates these findings (*z*-scored on baseline distribution to facilitate interpretation).

### Aim 3: To Quantify the Effects of Worry, Risk Perception, and Social Distancing on Well-being

More worry about health and financial consequences related to the COVID-19 pandemic were related to lower scores on life satisfaction (β = −0.12/−0.17, *p* < .001), financial satisfaction (β = −0.10/−0.25, *p* ≤ .001), and self-rated health (β = −0.16/−0.08, *p* ≤ .008), and higher scores on loneliness (β = 0.09/0.13, *p* ≤ .007). On the contrary, more worry about societal consequences was related to higher financial satisfaction (β = 0.09, *p* = .003) and less loneliness (β = −0.08, *p* = .009). Higher scores on social distancing were related to higher satisfaction with life (β = 0.11, *p <* .001) and finances (β = 0.08, *p* = .006). Societal risk and perceived risk of being infected were not significantly related to any of the four outcome variables (all *p* > .0125). For all estimates from the linear mixed-effects models, see Supplementary Table S3.

## Discussion

This study investigated the effects of the COVID-19 pandemic on well-being in a Swedish sample of older adults. Our first aim was to determine the level of worry, risk perception, and social distancing in response to COVID-19. Older adults aged 65–71 perceived high societal risks related to the COVID-19 pandemic; the majority reported having reduced close physical contact with others (social distancing), and were concerned about adverse effects on social structures and the world economy. At the same time, the majority were not particularly worried about their personal economy, nor for themselves or loved ones being infected by the virus; they perceived the risk of infection for themselves or loved ones as rather low. However, those who were given stricter recommendations (in our sample those 70 and older) regarded their risk of becoming infected as lower than those 65–69 years old. This might be explained by a higher degree of social distancing among those 70 and older, thus reducing the risk of infection. In sum, few participants reported being personally affected, while the majority generally considered COVID-19 a major threat to health, security, and well-being in Sweden and worldwide (Drury et al., 2013). The stricter recommendations to those 70 or older seem to have resulted in more social distancing in this group, but not more worry (Ayalon et al., 2020).

Our second aim was to investigate the longitudinal effects of the COVID-19 pandemic on well-being. Contrary to the expected negative impact of the pandemic (WHO, 2020c), across a 5-year period, we found no negative effect of COVID-19 on well-being. On the contrary, self-rated health was as high as 5 years ago, and financial satisfaction was higher than in any of the previous years. To illustrate these numbers, in 2019, 60% of participants rated their health as good or very good, while the corresponding number in 2020 was 69%. In 2019, 70% were satisfied or very satisfied with their financial situation, while in 2020 75% were satisfied or very satisfied. Life satisfaction and loneliness showed no deviation in 2020 compared to previous years. Although subjective health declines with increasing age, in 2020 that trend was broken, and subjective health was rated as high as in 2015. Based on these findings, we conclude that COVID-19, so far, has had few adverse effects on well-being among older adults in Sweden. Instead, most people rated their well-being just as high as or even higher than they did in previous years.

One possible explanation for this positive effect is a hesitance among older adults in Sweden to identify as “older,” as there have been reports of older citizens rejecting the notion that they belong to a risk group (Ekroth, 2020). However, this seems to be contradicted by our data showing high levels of social distancing, increasing with age. Another possible explanation is the effect of contrasts (Tversky, 1977). Circumstances that might have seemed less satisfying last year now seem more satisfying compared to the potential negative effects of COVID-19. Further, it is possible that the increased well-being in our sample is due to the contrast between the relative freedom of movement in Sweden and other countries’ strict regulations (Schwarz & Strack, 1999).

Our third aim was to quantify the effects of level of worry, risk perception, and social distancing on well-being. While levels of worry were generally moderate, COVID-19 may have caused some participants to worry to an extent that negatively affected their well-being. Those who worried more about negative health and financial consequences reported lower well-being, indicating that socioeconomic dimensions might influence the effects of the pandemic (Cappelen et al., 2020). Surprisingly, worrying more about adverse effects on social structures and the world economy were connected to higher well-being. Lastly, those who practiced more social distancing reported higher satisfaction with life and finances. Overall, this suggests that while many older adults handle the distress of the pandemic well, older adults are a heterogeneous group, and some older adults worry to the extent that their well-being suffers (Losada-Baltar et al., 2020).

Important limitations include the short time frame of data collection, the narrow age range of the participants, and potential problems in isolating worry and behavior caused by the pandemic from other events and circumstances. The 2020 data used in this study were collected during 1 week in the early days of the pandemic’s progression in Sweden and continued follow-ups are needed to evaluate if the effects persist over time. However, the time frame is also one of the foremost strengths of this study, as we have captured effects that later studies will miss. A major strength of the study is the HEARTS database, which presents a unique opportunity to analyze changes longitudinally.

In sum, Swedish older adults were still “up and about” during the early part of the COVID-19 pandemic; while the majority practiced social distancing, they also rated their well-being as high as, or even higher than they did 5 years prior. Generally low levels of worry and high well-being might be a consequence of the relatively few restrictions in Sweden, but studies from other countries will be needed for comparison. It is also important to note that older adults are a heterogeneous group; while most had high well-being, those who worried more about the health and financial effects of COVID-19 had lower well-being. Hence, determining ways to reduce worry will be important to mitigate lower well-being during the pandemic. Finally, the findings in this study should not be taken as an endorsement of any particular governmental response. Although subjective well-being is important, it is only one component of health. Older adults’ high well-being in this study should not be used to disregard other components of health. Hence, more research, on a broad range of health indicators, is needed to monitor and counteract the consequences of COVID-19.

## Supplementary Material

gbaa084_suppl_Supplementary_Material

## Data Availability

This data can be made available upon request and in accordance with applicable laws. For further information about accessibility of data, contact hearts@psy.gu.se

## Preregistered

This study was not preregistered.
