# Supplementary material for: Up and About: Older Adults’ Well-being During the COVID-19 Pandemic in a Swedish Longitudinal Study
Source: J Gerontol B Psychol Sci Soc Sci. 2020 Jun 30;76(2):e4–9. doi: 10.1093/geronb/gbaa084 (PMC7337833; doi:10.1093/geronb/gbaa084)
Supplement: gbaa084_suppl_Supplementary_Material [file gbaa084_suppl_supplementary_material.docx]

*Supplementary Table S1.*

Measurement details of the study variables.

| **Measure** | **Items** | **Scale range** | **Cronbach’s alpha / item correlation** |
| --- | --- | --- | --- |
| Life satisfaction^a^ (W1–6) | In most ways my life is close to my ideal | 1 (strongly disagree) to 7 (strongly agree) | .92–.93 |
|  | The conditions of my life are excellent |  |  |
|  | I am satisfied with my life |  |  |
|  | So far I have gotten the important things I want in life |  |  |
|  | If I could live my life over, I would change almost nothing |  |  |
| Financial satisfaction (W2–6) | How satisfied are you currently with your financial situation? | 1 (very dissatisfied) to 5 (very satisfied) | – |
| Self-rated health (W1–6) | How do you currently evaluate your overall health condition? | 1 (very bad) to 6 (very good) | – |
| Loneliness^ab^ (W1–6) | I lack companionship | 1 (strongly disagree) to 4 (strongly agree) | .76–.79 |
|  | I feel part of a group of friends |  |  |
|  | I feel left out |  |  |
|  | I feel isolated from others |  |  |
| Health worry^a^ (W6) | I worry about being infected and getting sick | 1 (strongly disagree) to 5 (strongly agree) | .63 |
|  | I worry that people close to me will be infected and get sick |  |  |
| Societal worry^a^ (W6) | I worry about the consequences on important societal functions | 1 (strongly disagree) to 5 (strongly agree) | .66 |
|  | I worry about the consequences for the economic development in society |  |  |
| Financial worry^a^ (W6) | I worry about the financial consequences for myself/my household | 1 (strongly disagree) to 5 (strongly agree) | .74 |
|  | I worry about the effects on my pension income |  |  |
| Societal risk^a^ (W6) | The coronavirus is a major risk to people’s health, security and wellbeing in Sweden | 1 (strongly disagree) to 5 (strongly agree) | .88 |
|  | The coronavirus is a major risk to people’s health, security and wellbeing in the world |  |  |
| Risk of being infected (W6) | How do you perceive your likelihood of getting infected by the coronavirus? | 1 (very unlikely) to 4 (very likely) | – |
| Social distancing^a^ (W6) | I avoid contact with others to protect myself from being infected | 1 (strongly disagree) to 5 (strongly agree) | .74 |
|  | I avoid contact with others to minimize spread |  |  |
| ^a^Scores averaged across items.  ^b^Item 2, 5, 11, and 14 from the UCLA Loneliness Scale. | | | |

*Supplementary Table S2.*

Bivariate correlations among the four wellbeing measures in the 2020 wave.

| **Variable** | **Life satisfaction** | **Financial satisfaction** | **Self-rated health** | **Loneliness** |
| --- | --- | --- | --- | --- |
| Life satisfaction | 1.00 | .40 | .51 | -.49 |
| Financial satisfaction | .40 | 1.00 | .28 | -.24 |
| Self-rated health | .51 | .28 | 1.00 | -.29 |
| Loneliness | -.49 | -.24 | -.29 | 1.00 |

*Supplementary Table S3.*

Estimates from linear mixed-effects models predicting life satisfaction, financial satisfaction, self-rated health, and loneliness.

|  | **Life Satisfaction** | | | | **Financial Satisfaction** | | | | **Self-Rated Health** | | | | **Loneliness** | | | |
| --- | --- | --- | --- | --- | --- | --- | --- | --- | --- | --- | --- | --- | --- | --- | --- | --- |
| **Parameter** | ***b*** | **SE** | ***p*** | **β** | ***b*** | **SE** | ***p*** | **β** | ***b*** | **SE** | ***p*** | **β** | ***b*** | **SE** | ***p*** | **β** |
| *Model 1* (*n*/obs.) | 1057/6031 | | | | 1057/5084 | | | | 1057/6139 | | | | 1057/6032 | | | |
| Intercept | 3.36 | 1.12 | .003 | 0.06 | 1.83 | 0.76 | .017 | 0.02 | 3.58 | 0.74 | <.001 | -0.23 | 1.98 | 0.50 | <.001 | -0.002 |
| Slope | 0.02 | 0.01 | .015 | 0.01 | 0.002 | 0.01 | .791 | 0.002 | -0.05 | 0.01 | <.001 | -0.05 | -0.002 | 0.004 | .690 | -0.003 |
| Age | 0.02 | 0.02 | .163 | 0.04 | 0.03 | 0.01 | .006 | 0.07 | 0.01 | 0.01 | .223 | 0.03 | -0.01 | 0.01 | .357 | -0.02 |
| Gender (female=1) | 0.05 | 0.07 | .514 | 0.02 | -0.18 | 0.05 | <.001 | -0.10 | 0.01 | 0.05 | .861 | 0.004 | -0.09 | 0.03 | .004 | -0.08 |
| Education (tertiary=1) | 0.14 | 0.07 | .045 | 0.05 | 0.17 | 0.05 | <.001 | 0.09 | 0.19 | 0.05 | <.001 | 0.10 | -0.01 | 0.03 | .664 | -0.01 |
| Retirement status (retired=1) | 0.18 | 0.03 | <.001 | 0.06 | -0.11 | 0.03 | <.001 | -0.05 | 0.12 | 0.02 | <.001 | 0.05 | -0.04 | 0.02 | .003 | -0.03 |
| 2020 (vs. previous years) | 0.03 | 0.03 | .324 | 0.02 | 0.08 | 0.03 | .003 | 0.09 | 0.16 | 0.03 | <.001 | 0.18 | -0.003 | 0.02 | .841 | -0.01 |
| *REML criterion at convergence* | 15103.5 | | | | 10724.7 | | | | 13135.1 | | | | 7065.0 | | | |
|  |  |  |  |  |  |  |  |  |  |  |  |  |  |  |  |  |
| *Model 2* (*n*/obs.) | 1028/5867 | | | | 1028/4942 | | | | 1028/5969 | | | | 1028/5869 | | | |
| Intercept | 4.38 | 1.14 | <.001 | 0.07 | 2.83 | 0.76 | <.001 | 0.03 | 3.73 | 0.77 | <.001 | -0.24 | 1.77 | 0.52 | <.001 | 0.003 |
| Slope | 0.02 | 0.01 | .012 | 0.01 | 0.004 | 0.01 | .664 | 0.004 | -0.05 | 0.01 | <.001 | -0.05 | -0.001 | 0.004 | .742 | -0.002 |
| Age | 0.01 | 0.02 | .656 | 0.01 | 0.02 | 0.01 | .177 | 0.03 | 0.02 | 0.01 | .173 | 0.03 | -0.003 | 0.01 | .745 | -0.01 |
| Gender (female=1) | 0.09 | 0.07 | .180 | 0.04 | -0.16 | 0.05 | <.001 | -0.08 | 0.05 | 0.05 | .315 | 0.03 | -0.10 | 0.03 | .002 | -0.09 |
| Education (tertiary=1) | 0.09 | 0.07 | .194 | 0.04 | 0.14 | 0.05 | .003 | 0.07 | 0.16 | 0.05 | <.001 | 0.09 | -0.003 | 0.03 | .927 | -0.002 |
| Retirement status (retired=1) | 0.18 | 0.03 | <.001 | 0.06 | -0.11 | 0.03 | <.001 | -0.05 | 0.12 | 0.02 | <.001 | 0.05 | -0.04 | 0.02 | .006 | -0.03 |
| 2020 (vs. previous years) | 0.03 | 0.03 | .389 | 0.02 | 0.08 | 0.03 | .004 | 0.09 | 0.17 | 0.02 | <.001 | 0.18 | -0.004 | 0.02 | .786 | -0.01 |
| Health worry | -0.13 | 0.04 | <.001 | -0.12 | -0.09 | 0.03 | .001 | -0.10 | -0.13 | 0.03 | <.001 | -0.16 | 0.05 | 0.02 | .007 | 0.09 |
| Societal worry | 0.08 | 0.04 | .055 | 0.06 | 0.09 | 0.03 | .003 | 0.09 | 0.01 | 0.03 | .664 | 0.01 | -0.05 | 0.02 | .009 | -0.08 |
| Financial worry | -0.17 | 0.03 | <.001 | -0.17 | -0.18 | 0.02 | <.001 | -0.25 | -0.06 | 0.02 | .008 | -0.08 | 0.06 | 0.01 | <.001 | 0.13 |
| Societal risk | -0.03 | 0.05 | .622 | -0.02 | 0.04 | 0.03 | .228 | 0.04 | 0.05 | 0.03 | .188 | 0.04 | -0.01 | 0.02 | .802 | -0.01 |
| Risk of being infected | 0.07 | 0.05 | .172 | 0.04 | 0.04 | 0.03 | .272 | 0.03 | 0.04 | 0.03 | .298 | 0.03 | -0.01 | 0.02 | .669 | -0.01 |
| Social distancing | 0.14 | 0.04 | <.001 | 0.11 | 0.08 | 0.03 | .006 | 0.08 | 0.001 | 0.03 | .969 | 0.001 | -0.04 | 0.02 | .026 | -0.07 |
| *REML criterion at convergence* | 14631.9 | | | | 10327.1 | | | | 12775.1 | | | | 6951.9 | | | |
